# Assessing the Quality of Care for Pneumonia in Integrated Community Case Management: A Cross-Sectional Mixed Methods Study

**DOI:** 10.1371/journal.pone.0152204

**Published:** 2016-03-24

**Authors:** Chomba Sinyangwe, Kirstie Graham, Sarala Nicholas, Rebecca King, Samuel Mukupa, Karin Källander, Helen Counihan, Mark Montague, James Tibenderana, Prudence Hamade

**Affiliations:** 1 Malaria Consortium, Mansa, Luapula Province, Zambia; 2 Malaria Consortium, London, United Kingdom; 3 The Nuffield Centre for International Health & Development, University of Leeds, Leeds, United Kingdom; 4 Malaria Consortium, Kampala, Uganda; Ohio State University College of Medicine, UNITED STATES

## Abstract

**Background:**

Pneumonia is the leading infectious cause of mortality in children under five worldwide. Community-level interventions, such as integrated community case management, have great potential to reduce the burden of pneumonia, as well as other diseases, especially in remote populations. However, there are still questions as to whether community health workers (CHW) are able to accurately assess symptoms of pneumonia and prescribe appropriate treatment. This research addresses limitations of previous studies using innovative methodology to assess the accuracy of respiratory rate measurement by CHWs and provides new evidence on the quality of care given for children with symptoms of pneumonia. It is one of few that assesses CHW performance in their usual setting, with independent re-examination by experts, following a considerable period of time post-training of CHWs.

**Methods:**

In this cross-sectional mixed methods study, 1,497 CHW consultations, conducted by 90 CHWs in two districts of Luapula province, Zambia, were directly observed, with measurement of respiratory rate for children with suspected pneumonia recorded by video. Using the video footage, a retrospective reference standard assessment of respiratory rate was conducted by experts. Counts taken by CHWs were compared against the reference standard and appropriateness of the treatment prescribed by CHWs was assessed. To supplement observational findings, three focus group discussions and nine in depth interviews with CHWs were conducted.

**Results and Conclusion:**

The findings support existing literature that CHWs are capable of measuring respiratory rates and providing appropriate treatment, with 81% and 78% agreement, respectively, between CHWs and experts. Accuracy in diagnosis could be strengthened through further training and the development of improved diagnostic tools appropriate for resource-poor settings.

## Introduction

Pneumonia accounts for 15% of all childhood deaths and kills more children than any other infectious disease worldwide, with an estimated 935,000 deaths of children under five per year attributable to this infection in 2013 [1]. The World Health Organization (WHO) and UNICEF estimate that through implementation of several key interventions, a substantial proportion of childhood pneumonia deaths could be averted [2]. The Global Action Plan for Prevention and Control of Pneumonia [2] outlines these key interventions; case management at all levels (including community); vaccination; prevention and management of HIV infection; improvement of nutrition and reduction of low birth weight; and control of indoor air pollution.

Community-level interventions have an important contribution to make through improving accessibility, uptake and appropriate use of services. Evidence has shown that pneumonia case management alone of infants and preschool children by community health workers (CHWs) resulted in a 36% reduction in pneumonia mortality [3]. However, the necessity of targeting not only pneumonia but also other important causes of childhood illnesses (especially malaria and diarrhoea) in an integrated way has also been recognised [3, 4]. Additionally, within the community it has been shown that increasing the number of illnesses CHWs treat increases demand for their services [5].

One outcome of the growing emphasis on community-level interventions has been the integrated community case management (iCCM) strategy for malaria, pneumonia, diarrhoea in children under five years of age, sometimes including newborn health and nutrition [6]. iCCM is currently being rolled-out in several countries across Asia, South America and sub-Saharan Africa. The overall aim of iCCM is to support and strengthen community-based case management in hard-to-reach communities by providing free diagnosis and treatment to children under five years old, in addition to training, job aids and supervision of CHWs. The iCCM package also includes behaviour change communication to caregivers and the wider community.

Interventions, such as iCCM, have great potential to increase access to health care and reduce the burden of pneumonia, especially in marginalised populations [6, 7]. However, increasing access to medicines must be implemented in parallel with equivalent efforts to understand and address the potential implications, which include ensuring children receive appropriate treatment for their condition, as well as defining the potential risk of contributing towards the development of drug resistance. Despite existing evidence, there remains an ongoing debate regarding the ability of CHWs to accurately diagnose the signs and symptoms of suspected pneumonia and provide appropriate treatment [8]. Evidence on CHWs’ ability is still quite limited, with studies often having significant methodological constraints. In particular, most have been conducted in formal health care settings and not in the CHW’s usual place of work [9–11]. This approach has the potential to introduce considerable bias and brings into question the extent to which findings are transferable to the community setting.

This research assessed the accuracy of respiratory rate measurement by CHWs using innovative methodology, and evaluated their ability to appropriately prescribe treatment for children with suspected pneumonia. It builds on previous studies to inform future implementation of iCCM, evaluating CHW performance in their usual context, with independent re-examination by experts and after a significant period of time post training [9, 12–15]. The research presented in this manuscript is one component of a mixed methods cross-sectional study that overall aimed to identify the percentage of CHWs who adhered to iCCM guidelines. Data exploring the rational use of antibiotics will be presented in a separate manuscript.

## Materials and Methods

### Study setting

Data was collected in Samfya and Kawambwa districts of Luapula province, Zambia between October and December 2012. Kawambwa and Samfya have a population of 130,680 and 191,980 and are in the north and south of the province, respectively [16].

### Study design

1,497 CHWs’ consultations, conducted by 90 CHWs over six weeks, were directly observed by nine non-clinical researchers in the CHW’s usual place of work, with measurement of respiratory rate by CHWs for 698 children recorded by video. The video camera was mounted on a tripod and placed in a discreet position prior to consultations. Researchers were instructed to record the measurement of respiratory rate by video for all children for whom the CHW conducted a respiratory rate count i.e. only those with suspected pneumonia. According to CHWs’ training, the respiratory rate of a child should be measured using an acute respiratory infection (ARI) timer in all children presenting with cough or difficulty in breathing. For children 2–11 and 12–59 months, a respiratory rate of ≥50 and ≥40 indicates suspected pneumonia, respectively. Researchers visited ten CHWs each, spending three days with each CHW. To ensure there were sufficient numbers of children with suspected pneumonia attending the CHW during the two day observation period, the community was sensitised during the first day to encourage them to bring any sick children under five years of age for treatment during the subsequent two days. Video footage was later used to conduct a retrospective reference standard assessment of respiratory rate by two pairs of experts. Experts were national-level certified master trainers in Integrated Management of Childhood Illnesses (IMCI) in Zambia. Each pair of experts viewed the video footage together, but recorded the respiratory rate count independently, and subsequently compared results. An average count was taken for expert counts within five breaths of each other. An inter-expert count variation of more than five prompted a re-assessment. For the most part, experts were blinded to the CHW count, as the respiratory rate measured by the CHW was recorded as a separate video clip following the initial recording. Three focus group discussions (FGDs) and nine in depth interviews (IDIs) with CHWs were also conducted to supplement observational findings. Researchers were all Zambian nationals from Luapula province with mixed experience of conducting research. However, all researchers took part in seven days of training, delivered in two parts, to ensure they had sufficient understanding to conduct the study. This included a detailed discussion on the appropriate translation of each of the questions in the semi-structured interview guides. All qualitative study components were audio-recorded, conducted in the local language (Bemba), and subsequently translated and transcribed into English. The IDIs were transcribed verbatim and the FGDs were transcribed using the Fairnotes approach [17]. The CHWs who were selected as interview respondents were asked to participate at the end of the second day of observation. The FGDs were held once the observation component of the study was completed for all 90 CHWs.

### Sample size

The sample size for the observation of CHWs was calculated to identify the proportion of CHWs who adhere to iCCM guidelines. A final sample size of 90 CHWs was calculated, assuming 50% of CHWs would be adherent to iCCM guidelines, with 95% confidence, at a precision of 10%, and including an adjustment of 10% to account for CHW non-response and data excluded from final analysis for any reason.

### Sampling

CHWs were selected for observation by simple random sampling from the total number (N = 440) of CHWs trained in iCCM in the two study districts, as part of the iCCM programme in Luapula Province funded by the Canadian International Development Agency (CIDA) from 2009 to 2013. CHWs had received training on iCCM at least one year prior to the study. For the IDIs, nine CHWs in total were systematically sampled, with the eighth CHW observed by each researcher invited to participate. For the three FGDs, 24 CHWs were selected to participate by convenience sampling from all those observed in the observation component of the study.

### Ethics

This study was approved by the School of Medicine Research Ethics Committee at the University of Leeds and the University of Zambia Biomedical Research Ethics Committee. Written consent was obtained from each CHW prior to the observation period, and from each caregiver of children under five who presented during the two days. Additional written consent was obtained from the CHWs who were subsequently selected for the IDIs, and those who participated in the FGDs.

### Data analysis

#### Quantitative analysis

All quantitative data were analysed using Stata version 12 (StataCorp LP). Of the 1,497 consultations that were observed, seven (<0.005%) were excluded due to: no data being recorded (n = 2), child was aged more than five years (n = 1), or if the child’s age was not specified (n = 4). 698 videos were recorded where the CHW measured the respiratory rate of the child. Of this number, 537 videos (77%) were included in the analysis and reviewed by experts, to compare the counts taken by the CHW against a reference standard. 161 videos (23%) were excluded for various reasons that included poor video quality, missing information or child was breastfeeding, crying during measurement of the respiratory rate or where the expert ratings of respiratory rate differed by more than five counts and had not been recounted.

Descriptive results are summarised as frequencies, percentages or as a median with inter-quartile range (IQR). Indicators are presented as percentages with 95% confidence intervals (CI) adjusting for clustering of children by CHW. Pearson chi-squared test accounting for clustering by CHW was used to assess association between each indicator and age group of the children.

Scatterplots and Bland-Altman plots [18] were produced to assess the agreement in ratings of respiratory counts between experts and CHWs. The Bland-Altman method plots the difference in ratings (y-axis) against the average of the ratings (x-axis) for every child observed. Superimposed upon these figures is the mean difference in ratings between experts and CHWs (the bias) and the 95% limits of agreement defined as +/-2 standard deviations of the bias. These limits indicate a plausible range of values where 95% of the observed differences should lie. The narrower the range of values, the better the agreement. Levels of significance were obtained by the adjusted Wald test. Furthermore, the proportion of respiratory rate counts by CHWs within five, three and two breaths of the expert count was calculated.

Children were classified as having fast breathing if they presented with a respiratory rate of more than or equal to 50 breaths per minute or 40 breaths per minute for younger (2–11 months) and older children (12–59 months) respectively. Children with normal breathing had respiratory rates below these cut-off points. Cohen’s Kappa was obtained to assess the level of agreement between the classification of fast and normal breathing by experts and CHWs. Sensitivity and specificity analyses were also performed, to determine the proportion of children who were correctly classified as having fast or normal breathing.

Fast breathing in children with cough or difficulty breathing is indicative of suspected pneumonia and should be treated with antibiotics, as per the iCCM guidelines. The sensitivity and specificity of treatment given by the CHW was calculated. Sensitivity and specificity indicates the proportion of children observed who were correctly prescribed antibiotics for fast breathing, as assessed by an expert, and the proportion of children observed who had normal breathing and were not given antibiotics, respectively. Cohen’s Kappa was calculated to assess overall agreement of treatment given, indicating the proportion of children observed who were appropriately treated, receiving antibiotics for fast breathing and not receiving antibiotics for normal breathing, as assessed by an expert.

#### Qualitative data analysis

Qualitative data was managed using NVivo 10 (QSR International) and analysed by thematic analysis. The coding frame consisted of both a priori and grounded codes, and was developed and agreed by RK, KG and CS based on an initial analysis of three IDI transcripts. The remainder of the transcripts were analysed by KG, with any changes to the coding frame or queries discussed with RK and CS as necessary. Summaries of each theme were also reviewed by each researcher before data was consolidated and written up. Unfortunately, it was not possible for the study participants to review final transcripts or provide feedback on study findings.

## Results

### Participants

The median age of the 90 CHWs observed was 44 years (IQR: 38–49) and 19% were female. The majority (77%) had attained a secondary education and 90% worked in the same community as from where they originated. 99% of CHWs reported that they had worked for more than one year as a CHW and 44% reported that they had worked more than five years as a CHW. All participants had received the six-day iCCM training supported by Malaria Consortium as part of the CIDA-funded implementation of iCCM in Luapula Province. However, 79% of the CHWs had also previously received six-week CHW training from the Ministry of Health. This proportion increased to 87% if CHW and community IMCI training were considered. The median number of types of training received was three and only one CHW reported having no additional training.

The median number of children observed per CHW was 14 (IQR: 10–21) during the two days of observation. 50% of children observed were male and 25% were less than 12 months of age (Table 1). The characteristics of all children were similar, regardless of whether or not the measurement of respiratory rate was recorded on video.

**Table 1:** Characteristics of children observed.

	**All children (N = 1490)**		**Children on video (N = 537)**		**Other (N = 952)**	
**Child’s age**	**n**	**%**	**n**	**%**	**n**	**%**
**2–11 months**	368	25	147	27	221	23
**12–59 months**	1122	75	390	73	732	77
	**All children (N = 1486)**		**Children on video (N = 535)**		**Other (N = 951)**	
**Child’s sex**	**n**	**%**	**n**	**%**	**n**	**%**
**Male**	739	50	260	48	479	50
**Female**	747	50	275	51	472	50
	**All children (N = 1480)**		**Children on video (N = 536)**		**Other (N = 944)**	
**Guardian**	**n**	**%**	**n**	**%**	**n**	**%**
**Mother**	1380	93	503	94	877	93
**Father**	55	4	21	4	34	4
**Other**	46	3	12	2	34	4

There were nine in depth interviews conducted with CHWs, seven were male, one was female, and the gender of one participant was not recorded by the researcher. The FGDs involved 24 CHWs, consisting of seven females and 17 males, with eight participants per group. IDIs and FGDs were approximately 40 minutes and two hours in length, respectively.

### Assessment of respiratory rate in children: comparison of CHW and expert respiratory rate counts

**Fig 1:**
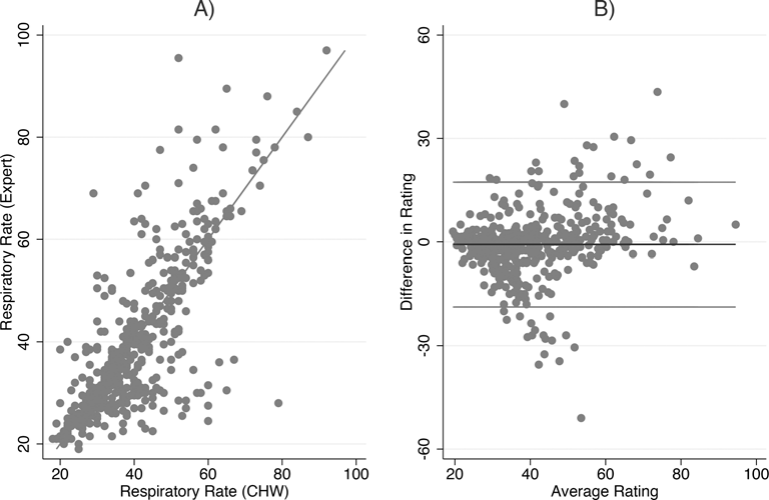
Relationship between expert and CHW respiratory count for all children. (A) Scatterplot, (B) Bland Altman plot.

Overall, there is a strong correlation between expert and CHW respiratory counts (Pearson correlation coefficient = 0.78) (Fig 1A). The mean difference in respiratory rate counts between experts and CHW (bias) as obtained by the Bland-Altman method (Fig 1B) was -0.74 (95% CI: -1.96 to 0.49), which is not statistically different from 0 (Adjusted Wald Test, p = 0.23). However, the variability (as shown by the scatter around the mean difference) is not consistent; while CHWs’ counts are in general higher than those of experts, when average respiratory counts are greater than 60, CHWs’ counts are lower than experts. Further, the standard deviation of the mean difference is large (SD = 9.0) giving a wide limits of agreement between -18.8 to 17.3. Examining the percentage of CHW respiratory counts that are within two, three or five breaths of expert count showed 46% (95% CI: 39 to 53) were within two breaths and 55% (95% CI: 46 to 63) and 67% (95% CI: 58 to 75) were within three or five breaths respectively.

When examined by age group, the correlation between respiratory rate counts by experts and CHWs is also high at 0.74 and 0.70 for children aged 2–11 months and 12–59 months respectively. The mean difference in ratings for children aged 2–11 months is 2.04 (95% CI: 0.02 to 4.06) and -1.77 (95% CI: -2.92 to -0.64) for 12–59 months. Both are significantly different from 0 (Adjusted Wald Test, p = 0.05 and p = < 0.01, respectively). For children aged 2–11 months, the data implies that CHWs consistently scored respiratory rates lower than experts compared with the older age group, where it suggests that CHWs consistently scored respiratory rates higher than experts. For both groups, there is inconsistent and wide variation in the difference in ratings, with the limits of agreement lying between -19.7 and 23.8 for the younger group and -17.7 and 14.2 for children aged 12–59 months.

In terms of the percentage of CHW respiratory counts that were within two, three or five breaths of experts, for the younger age group, this equates to 40% (95% CI: 32 to 49), 50% (95% CI: 40 to 60) and 61% (95% CI: 51 to 71) respectively. For the older age group, 48% (95% CI: 40 to 57), 56% (95% CI: 48 to 65) and 69% (95% CI: 60 to 77) were within two, three or five breaths respectively.

In terms of fast breathing, 40% of children (213/537) were classified as having fast breathing by CHW compared to 33% (180/537) by experts. Both CHWs and experts found a higher percentage of children with fast breathing in the younger age group (p = < 0.01). CHWs diagnosed 54% of children aged 2–11 months to have fast breathing compared to 34% of children aged 12–59 months. Experts diagnosed 56% of children aged 2–11 months to have fast breathing compared to 25% of children aged 12–59 months.

CHWs were 81% in agreement with experts when classifying children as having fast or normal breathing, with a sensitivity and specificity of 81% and 81%, respectively (Table 2). Similar agreement was found amongst younger and older children, however sensitivity was slightly lower when CHWs diagnosed older children. Cohen’s Kappa statistic ranged between 0.54 to 0.63 denoting moderate agreement.

**Table 2:** CHW classification of fast and normal breathing compared to expert classification.

	All children		Children 2–11 months		Children 12–59 months	
	Expert		Expert		Expert	
CHW	Fast breathing	Normal breathing	Fast breathing	Normal breathing	Fast breathing	Normal breathing
**Fast breathing**	145	68	67	12	78	56
**Normal breathing**	35	289	15	53	20	236
**Agreement (95% CI)**	81 (76–85)		82 (73–88)		81 (75–85)	
**Kappa (κ) value**	0.59		0.63		0.54	
**Sensitivity (95% CI)**	81 (73–87)		82 (69–90)		80 (70–87)	
**Specificity, (95% CI)**	81 (75–86)		82 (70–89)		81 (74–86)	

When the participants of the FGDs were asked to describe how a child with pneumonia would be diagnosed, one or two participants took the lead and described the process in depth. Whilst the others were strongly in agreement, it does not give a representative assessment of knowledge within the group, only of one or two individuals.

However, the knowledge of those who responded was quite high and followed the key steps as outlined in their training materials. All three of the CHW focus groups noted the need to count the respiratory rate or the number of breaths to diagnose suspected pneumonia, and the use of an ARI timer. Furthermore, all groups detailed correctly the different categories for fast breathing depending on the age of the child. Two of the focus groups also mentioned asking the caregiver to hold the child still or to ensure that there is no movement, taking off the child's clothes, asking the age of the child and checking for chest in-drawing, which indicates the need for urgent referral. One of the focus groups also added the need to restart the timer if the child moves. Another specifically mentioned looking for fast breathing and counting the breaths for one minute.

The in depth interviews with individual CHWs also seemed to indicate a high level of knowledge regarding pneumonia diagnosis and assessment of respiratory rate. Some participants shared more detail than others, thus appearing more knowledgeable, however this is not necessarily indicative of a higher level of knowledge as these are qualitative assessments.

Nevertheless, it is interesting to note that of the CHWs who specified the age categories for fast breathing, all except one were correct. The CHW who was not correct simply said that "I count when the child breathes out and when the child breathes 35 per minute then I know that the child has pneumonia" (CHW IDI 6). Another CHW highlighted that "we know the difference in breaths per minute according to the child's age" (CHW IDI 7), but did not elaborate further with specific age groups or number of breaths. Furthermore, all of the CHWs mentioned that they would count the breaths of the child and the majority said they would use a timer, although one called it a "heart timer" (CHW IDI 1). A few further elaborated that they would count breaths for one minute.

On the other hand, there were a few who did not mention asking the age of the child, or defined age categories for fast breathing. Also, only a few individuals specifically mentioned chest indrawing, or referring to checking "how the patient is looking under the ribs" (CHW IDI 9). There were a couple of others, in addition to these few, who highlighted that they would need to remove the child's clothes "so that the stomach and chest are exposed" (CHW IDI 8), which is in line with the normal procedure for checking respiratory rate, but may also imply that they were looking for chest indrawing.

### Treatment of suspected pneumonia

Appropriate treatment is defined as receiving antibiotics for fast breathing, as assessed by an expert, and not receiving antibiotics for normal breathing. Analysis indicated that 78% of children were appropriately treated following measurement of their respiratory rate, indicating overall agreement (Table 3). There was no significant difference between the two age groups (p = 0.80). Cohen’s Kappa statistic was 0.51 indicating moderate agreement.

**Table 3:** Medication prescribed according to assessment of fast breathing by expert and child's age.

	All children		Children 2–11 months		Children 12–59 months	
	Expert		Expert		Expert	
Received antibiotics	Fast breathing	Normal breathing	Fast breathing	Normal breathing	Fast breathing	Normal breathing
**Yes**	131	71	62	14	69	57
**No**	49	286	20	51	29	235
**Agreement (95% CI)**	78 (73–82)		77 (67–84)		78 (72–83)	
**Kappa (κ) value**	0.51		0.54		0.47	
**Sensitivity (95% CI)**	73 (63–81)		76 (61–86)		70 (59–79)	
**Specificity (95% CI)**	80 (73–86)		78 (65–88)		81 (73–86)	

Based on the expert assessment of respiratory rate, the sensitivity of treatment prescribed by CHWs was 73% (Table 3). There was no significant difference (p = < 0.01) between the proportion of children with fast breathing, as assessed by an expert, who were correctly prescribed antibiotics between the two age groups. Specificity of treatment, with children with normal breathing not receiving antibiotics, was 80%.

## Discussion

The level of agreement found in this study between CHWs’ classification of children with fast and normal breathing, and expert classification (reference standard) (81%) is within the range shown in other studies of 75 to 85% [9, 12, 14, 15]. Table 4 presents a detailed comparison of the data collected in this study with existing literature. The findings from our study are comparable, taking into account the size of the study, location of observations, the duration of time post-training when the study took place and the methodology used to assess performance of CHWs.

**Table 4:** Comparison of data with literature on CHW performance in measuring respiratory rate.

Country	No of CHWs	No of consultations (total)	Method^a^	Time post-training	Location	Sensitivity (%)	Specificity (%)	Agreement (%)	κ-value	+/- 5 breaths (%)	+/- 3 breaths (%)	+/- 2 breaths (%)
Zambia	90	538	DO+RE	>1 year	Community	81	81	81	0.58	67	55	46
Malawi [12]	131	382	DO+RE	Max. 23 months	Community	59	82	77	0.35	-	-	-
Uganda [14]	57	-	DO+RR	-	Health facility	-	-	75	-	49	39	-
Uganda [9]	14	13 (182)	DO	3 days	Health facility	-	-	84.6	0.665	-	-	64
Uganda [15]	96	576	DO	2 days	Health facility	75	83	79	0.75	71	-	-
Bangladesh [13]	120	1,166	DO	64.5 months^b^	Community	67.7^c^	95.2^c^	89^c^	-	-	-	-

^a^ RR: register review, RE: re-examination, DO: direct observation

^b^ median experience

^c^ cases classified as: mild, severe, very severe

Whilst the mean difference of -0.74 in respiratory rate counts between experts and CHWs is not statistically significant from 0, the limits of agreement are wide and there is inconsistent variation in the differences over the range of measurements, showing poor agreement between CHWs and experts. However, it is worth noting that achieving strong agreement on respiratory counts is difficult, even between experts [19]. The findings suggest that there is a tendency for CHWs to measure rates closer to 60 per minute, regardless of whether the respiratory rate is high or low. This may be due to the fact that the ARI timer used to measure the respiratory rate ticks once per second, however, this finding was also reported by Noordam et al. [20] regardless of the device used to assess respiratory rate.

The data indicate that there is a significantly higher percentage of younger children with fast breathing, as documented in the literature [21]. Also, it implies that younger children are potentially harder to diagnose as the variability between expert and CHW respiratory rate counts is slightly greater, however the sensitivity and specificity for the classification of fast breathing were not affected.

The qualitative findings from FGDs and IDIs with CHWs also indicate that there is a good understanding of how to diagnose suspected pneumonia and measure respiratory rate, including the different cut-off values for fast breathing according to age.

The data presented here provides further evidence that CHWs are capable of assessing children with suspected pneumonia using the tools provided, but accuracy of respiratory rate measurement could be strengthened through further training and the development of improved diagnostic devices, such as revised respiratory rate counters, appropriate for use by CHWs in these remote contexts.

This study contributes new evidence to the existing literature on the quality of care provided for children with suspected pneumonia by CHWs at community level through iCCM. A number of studies have conducted assessments of CHWs’ ability to accurately measure and classify respiratory rates of children. However, these studies rarely evaluate CHWs’ performance in their day-to-day environment, without direct and immediate validation of respiratory rate by a clinician, and seldom after a considerable period of time has elapsed post-training [9, 13–15]. For example, in a study conducted by Mukanga et al.[9], 14 CHWs were directly observed at a health facility by a paediatrician and laboratory scientist, two weeks post training, as the objective was to assess CHW competence after training. It has been recommended that studies are conducted in “real life setting” to gain a more accurate picture of their normal performance level [9, 15]. Rowe et al.[22] found that CHWs were less prone to errors in a hospital setting, with direct or non-direct observation, compared with working unobserved in their usual context. Furthermore, as detailed by Cardemil et al.[12], direct observation with re-examination was found to be the most reliable and appropriate method for accurate assessment of CHW performance in diagnosing uncomplicated cough and fast breathing. This assessment of methods was conducted in the CHW’s day-to-day context, rather than a health facility, with the children involved representing a similar case mix to normal [12].

In our study, the observation of CHW consultations took place in the CHWs place of work and involved direct observation with re-examination. It is also hoped that the Hawthorne effect [23] of behaviour changing as a result of being observed, was minimised as only one non-clinical researcher was involved and was present with each CHW for three days, and expert assessment of the respiratory rate was conducted independently at a later date. Furthermore, research by Pringle and Stewart-Evans [24] suggests that the use of a video to observe consultations does not affect health worker performance, although it is acknowledged that these findings are from a developed country context with higher-level health workers. While our study involved slightly less CHWs than other studies, approximately 1,500 sick child consultations were observed in total, with measurement of respiratory rate recorded in 698. CHWs involved in the study had not received any formal training for at least one year, but did receive monthly supportive supervision from health facility staff.

When prescription of antibiotics is based on the reference standard classification of fast breathing, a high percentage of children (73%) were correctly prescribed antibiotics (sensitivity). This tends to be higher than is reported in literature, with 40% and 63% reported by Mukanga et al. and Cardemil et al., respectively [9, 12]. Specificity of treatment in our study was 80%, compared with 75% reported by Cardemil et al [12]. This high level of specificity is encouraging, and helps to address residual concerns regarding the use of antibiotics at community level and the risk of over prescription. Furthermore, considering both children with or without fast breathing, a high proportion were appropriately treated (78%) (overall agreement).

In conclusion, the findings of the study indicate that CHWs can provide appropriate treatment for children under five years old with suspected pneumonia, however further training and the development of improved or new diagnostic tools, such as pulse oximeters [25], for assessing symptoms of pneumonia for use by CHWs in remote settings in low and middle income countries could further assist the accuracy of diagnosis and the appropriateness of treatment provided.

This study has a number of limitations. In terms of sampling, CHWs located on islands in the swamps in Samfya were not included, as it was not logistically possible to reach them within the timeframe and resources available. Also, stockouts were not assessed during this study, so it is not possible to determine if these influenced treatment given by the CHWs. It is acknowledged that CHW’s overall classification of disease (i.e. pneumonia, malaria, diarrhoea) was not recorded as part of this study, only the measurement of respiratory rate and the subsequent action taken. The data presented here also does not intend to comment on CHWs overall identification and assessment of diagnosis of pneumonia symptoms (i.e. cough, chest indrawing). Furthermore, a large number of the videos collected needed to be excluded from the analysis, in part due to the challenges of collecting data within this context. In addition, it is appreciated that the results from the FGDs are not representative of all participants, as they tended to be led by a few participants, whilst others were less actively involved.
